# Foraging through emotions: emotional stimuli and participants’ trait anxiety shape visual foraging

**DOI:** 10.1098/rsos.242175

**Published:** 2025-05-21

**Authors:** Jérôme Tagu, Christelle Robert, Stéphanie Mathey

**Affiliations:** ^1^ Univ. Bordeaux, LabPsy, UR 4139, F-33000 Bordeaux, France

**Keywords:** visual foraging, attention, emotion, target selection, trait anxiety, visual search

## Abstract

Previous work suggests that target selection during visual foraging is achieved through competition between different factors (e.g. proximity, priming, value) that orient attention towards one of the possible targets. However, this research has mainly involved simple stimuli such as coloured dots. Here, we investigated whether target selection is sensitive to the emotional content of the stimuli during visual foraging, using real-world photographs eliciting negative, neutral or positive emotions. Moreover, based on results from single-target visual search, we examined how participants’ trait anxiety influences foraging behaviour. Seventy-five observers completed three foraging tasks corresponding to three emotional-valence conditions (positive, neutral, negative). The task was to select all the targets (pre-specified emotional images) as fast as possible while ignoring neutral distractors. Observers’ foraging strategy (i.e. selection order, number of switches between target types) and performance (i.e. selection times, number of distractor selections) were measured. We also assessed participants’ trait anxiety. The results revealed that negative emotional stimuli significantly influenced both foraging strategy and performance. Furthermore, the effect of negative emotion on foraging performance was amplified for participants with high trait anxiety. These findings suggest that emotional characteristics of both targets and participants contribute to target selection during visual foraging.

## 1. Introduction

During the last 40 years, visual search tasks have been the main tool for investigating which factors determine the allocation of visual attention in humans (for review, see [1]). Most of this research coincides with the idea that search behaviour is determined both by top-down (e.g. goal-driven) and bottom-up (e.g. stimulus-driven) mechanisms. Top-down factors include, e.g. the known characteristics of the target, such as its expected colour, shape or location, while bottom-up factors refer to properties of the visual environment that attract attention, such as stimuli with unique features or that are brighter or larger than other objects in the environment (e.g. [2–5]). It is worth noting that research on visual attention has for the most part involved simple stimuli such as coloured shapes or letters, which are not supposed to convey emotions. However, our visual environment is mostly composed of rich, complex and meaningful stimuli, some of them being emotion laden. Emotional stimuli can be characterized by two main dimensions: emotional valence and arousal (e.g. [6]). Emotional valence refers to the pleasure–displeasure elicited by the stimulus, while the degree of arousal refers to the intensity of the emotion. As an example, a picture of a snake is a visual stimulus evoking displeasure (negative valence), yet its arousal may fluctuate depending on whether the snake is asleep or awake [7]. During visual search, emotional stimuli are well known to capture attention over non-emotional stimuli [8–10]. In addition to the emotional characteristics of the stimuli, the analysis of individual differences in visual search performance reveals that individual characteristics, such as personality traits and trait anxiety, also influence search behaviour, beyond the known influences of the visual environment [5,11]. Yet, up to now, the influence of emotions on multiple-target visual foraging has never been examined, whether in terms of the emotion elicited by the stimuli or the emotional characteristics of the observers.

### 1.1. Visual search for emotional stimuli

Emotions play an important role in our everyday lives, and more and more research is being conducted to investigate the influence of emotional stimuli on cognitive activities. Emotional stimuli such as facial expressions have been shown to attract attention compared to other visual stimuli [12–15]. As an example, using a visual search task with faces as stimuli, Schubö *et al*. [14] showed that threatening faces among neutral or happy faces were found faster than neutral faces among threatening faces. Importantly, Lamy *et al*. [13] replicated these findings using both angry and happy faces as targets among neutral-face distractors, suggesting that the advantage of emotional targets during visual search is found both for positive and negative emotional valences.

Altogether, these results reveal an attentional bias towards emotional stimuli during visual search. In other words, emotional stimuli with positive or negative valence capture attention over neutral stimuli. Consequently, search performance (i.e. accuracy and reaction times) is enhanced for emotional targets and impaired for emotional distractors. Importantly, research has shown that this effect is not restricted to faces, but it also applies to other emotional visual stimuli such as images and photographs (for reviews, see [8,9]). Moreover, the recent meta-analysis by Zsidó [10] indicates that stimuli with high arousal attract attention regardless of their emotional valence, improving search performance for task-relevant targets and impairing performance for task-irrelevant distractors.

### 1.2. The influence of trait anxiety on visual search

Recent research suggests that in addition to the stimulus characteristics, individual characteristics, such as personality traits or trait anxiety, also influence visual attention (for a recent review, see [11]). Trait anxiety refers to the general tendency of an individual to experience persistent and/or intense anxiety [16,17]. The attentional control theory [18] posits that trait anxiety decreases attentional control, leading to impaired performance in tasks involving attention, particularly in the presence of threat-related stimuli (which, by definition, elicit negative emotional valence). It suggests that, when performing a cognitive task, anxious individuals are more sensitive to threat-related stimuli in a relatively automatic way so that they take longer to respond as compared to non-anxious individuals. This deleterious effect of anxiety on processing speed would not be observed on accuracy because of efficient compensation with additional effort.

In the context of visual attention, Moran & Moser [19] found that individuals with high trait anxiety showed slower reaction times in a visual search task involving a salient distractor. Moreover, their results on event-related potentials revealed that the N2pc component, a measure of attentional selection, was enhanced in participants with high trait anxiety when the salient distractor was displayed close to the target. In accordance with the attentional control theory, the authors suggest that individuals with high trait anxiety show deficits in inhibiting salient distractors and need more time and enhanced attentional control to attain similar search accuracy as other participants.

Importantly, the effects of trait anxiety on reaction times at visual search tasks are found with non-emotional stimuli but are enhanced in the presence of threatening emotional stimuli [11,18]. As an example, a number of studies report that individuals with high trait anxiety show increased reaction times at visual search tasks involving threatening distractors, even when they are not particularly salient [20–22]. Moreover, Byrne & Eysenck [23] found that high trait anxiety was associated with facilitated detection of threatening targets. Altogether, these studies suggest that high trait anxiety is associated with impaired disengagement from salient distractors and from emotional stimuli with negative valence [11,22].

### 1.3. Multiple-target search or visual foraging

Until this point, we have described research that involved visual search tasks where observers indicate on each trial whether a unique target is present or absent among several distractors. However, this may not be a very realistic model for everyday visual orientation since our goals are seldom so narrow as to involve only a single target. Recently, multi-target ‘foraging’ tasks have been used to assess orienting in the visual field (e.g. [24,25]), and the results have raised important challenges for theories of visual attention (for review, see [26]).

Kristjánsson *et al*. [24] assessed whether human foraging varies with task difficulty. Participants performed a foraging task on an iPad. They were required to select all the targets by tapping on them with their fingers in both a feature-based and a conjunction-based foraging task. In the former, targets were identified by their colour only (e.g. red and green target dots among yellow and blue distractor dots), whereas in the latter, targets were identified by a combination of their colour and shape (e.g. red-dot and green-square targets among green-dot and red-square distractors). The main findings were that participants chose to locate the nearest target during feature foraging and frequently switched between the two target types. However, they encountered difficulties doing so during conjunction foraging. Instead, they focused on a single target type until the entire category was exhausted before turning to the second target type (e.g. they selected all the red dots before turning to the green squares).

These foraging strategies can be assessed by counting the number of ‘runs’ a participant makes during a trial. A ‘run’ refers to the sequential selection of targets of the same category (with 40 targets from two categories, the number of runs ranges between 2 and 20). During feature foraging, participants typically foraged in numerous short runs, while during conjunction foraging, they typically foraged in two long runs [24]. These results suggest that humans adapt their selection strategy to the difficulty of the foraging task. For easy feature-based targets, they locate the nearest target and frequently switch between target types. By contrast, for more difficult conjunction-based targets, they seemingly maintain only one target type in working memory and forage in runs. Consistent with this idea, inter-target times, which correspond to the average time elapsed between two successive target selections, were also longer in conjunction foraging than in feature foraging.

Later studies identified other factors affecting foraging performance, such as time constraints [27,28], target prevalence in the display [29,30], and target value [30,31]. Tagu & Kristjánsson [31] examined whether target value and proximity could simultaneously influence foraging performance when they are manipulated conjointly. They used a feature-foraging paradigm where observers earned points when selecting targets. The task was to reach a pre-specified number of points as fast as possible by selecting targets, among distractors. The authors contrasted a value block (in which one of the target types was more valuable) with a no-value block (where all target types were worth the same number of points). Moreover, they varied target proximity by manipulating the spatial organization of the display. On half of the trials, items from one of the three target types were distributed into ‘patches’, while on the other half, all target categories were randomly distributed in the display. Importantly, the results on the number of runs and on inter-target times confirmed that both target value and target proximity influence foraging behaviour. Moreover, when both factors were pitted against each other, observers tended to prioritize value over proximity. In other words, on ‘patched’ trials, observers tended to select all the high-value targets first, even when doing so made them travel through distant patches of high-value targets while passing over quite a few of the low-value targets on the way. Tagu & Kristjánsson [31] suggest that this reflects a ‘selection balance’ where different factors (including but not limited to target value and proximity) compete to determine the observer’s next selection.

Importantly, in all studies described above, individual differences in foraging behaviour have been reported. Notably, in all studies contrasting feature and conjunction foraging, a subgroup of observers chose to continue locating the nearest target and switching between target types during difficult conjunction foraging (e.g. [24,32,33]). The factors driving these individual differences are, however, still unknown. Jóhannesson *et al*. [32] investigated whether they were related to individual characteristics such as higher working memory capacity or inhibitory control, but the results did not reveal any relationship between these factors and foraging strategy (assessed with the number of runs) or performance (assessed with inter-target times). Given the few studies dedicated to this issue, the links between individual differences in foraging behaviour and other individual characteristics remain to be examined. Based on the well-documented effect of trait anxiety on single-target visual search performance (e.g. [11]), it would be logical to address this issue in the field of multiple-target visual foraging, especially when emotional stimuli are used.

### 1.4. The current study

Research into the factors driving target selection during visual foraging is very recent, and further studies need to be carried out to identify new factors contributing to target selection. Notably, in the current study, we aim to examine the attentional bias towards emotional targets in the context of multiple-target visual foraging. Although the contribution of target emotional characteristics on single-target visual search performance is well documented, it has never been studied in the context of visual foraging. Yet, it may have differing behavioural consequences when searching for multiple targets in a rich visual environment composed of multiple targets and distractors. Indeed, when foraging for emotional targets, all targets in the display should attract attention and may act as distractors to the observer sequentially foraging through all the (emotional) targets in the display, among neutral distractors. We therefore hypothesize that foraging performance will be poorer when foraging for emotional targets compared with neutral targets.

Moreover, and based on the assumptions of the attentional control theory [18], we expect that the attentional bias towards emotional targets may differ as a function of the participants’ trait anxiety. We further hypothesize that individual differences in trait anxiety may predict individual differences in foraging strategy (e.g. on the number of runs) and performance (e.g. on inter-target times), particularly for emotional targets with negative valence. We expect that participants with high levels of trait anxiety will forage with fewer runs and higher inter-target times, especially when foraging for negative targets.

## 2. Methods

### 2.1. Participants

A total of 75 participants (54 females) aged from 18 to 38 years (mean age = 21.2, s.d. = 2.9) were recruited from the University of Bordeaux. All participants were French native speakers and reported normal or corrected-to-normal vision. Prior to their inclusion in the study, they received clear explanations about the procedure and gave their written informed consent. The experimental procedure was approved by the local ethics committee (authorization number 2023.02.CLE008) and complied with the ethical guidelines set out in the 1964 Declaration of Helsinki and its later amendments.

### 2.2. Materials

We used 12 images from the International Affective Picture System (IAPS) [7] as stimuli. The IAPS database is composed of real-world colour photographs depicting visual scenes from a wide range of categories, with valence ratings ranging from 1 (negative) to 9 (positive) and arousal ratings ranging from 1 (calm) to 9 (excited). In the current experiment, we selected images with negative valence (<3), positive valence (>7) and neutral valence (>4 and <6, i.e. close to the midpoint of 5). Negative- and positive-valence stimuli were matched on arousal (>6 for both conditions), while neutral images were lower in arousal (<4). Moreover, in an attempt of minimizing any perceptual saliency effect, we selected images with similar size (no black borders) and similar hue distribution (for each image, we plotted the pixel distribution in colour space and selected images with similar hue distributions for each set of images). In the end, we created three sets of four images for the current experiment:

— Negative set: two negative target images: IAPS images no. 3110 (BurnVictim; valence: M = 1.79, s.d. = 1.30; arousal: M = 6.7, s.d. = 2.16) and no. 9325 (Vomit; valence: M = 1.89, s.d. = 1.23; arousal: M = 6.01, s.d. = 2.54); and two neutral distractor images: IAPS images no. 2397 (Men; valence: M = 4.98, s.d. = 1.11; arousal: M = 2.77, s.d. = 1.74) and no. 7040 (DustPan; valence: M = 4.69, s.d. = 1.09; arousal: M = 2.69, s.d. = 1.93) with similar hue distributions.
— Positive set: two positive target images: IAPS images no. 8190 (Skier; valence: M = 8.10, s.d. = 1.39; arousal: M = 6.28, s.d. = 2.57) and no. 8200 (WaterSkier; valence: M = 7.54, s.d. = 1.37; arousal: M = 6.35, s.d. = 1.98); and two neutral distractor images: IAPS images no. 2411 (Girl; valence: M = 5.07, s.d. = 0.85; arousal: M = 2.86, s.d. = 1.84) and no. 5390 (Boat; valence: M = 5.59, s.d. = 1.54; arousal: M = 2.88, s.d. = 1.97) with similar hue distributions.
— Neutral set: two neutral target images: IAPS images no. 7000 (RollingPin; valence: M = 5.00, s.d. = 0.84; arousal: M = 2.42, s.d. = 1.79) and no. 7050 (HairDryer; valence: M = 4.93, s.d. = 0.81; arousal: M = 2.75, s.d. = 1.80); and two neutral distractor images: IAPS images no. 7004 (Spoon; valence: M = 5.04, s.d. = 0.60; arousal: M = 2.00, s.d. = 1.66) and no. 7010 (Basket; valence: M = 4.94, s.d. = 1.07; arousal: M = 1.76, s.d. = 1.48) with similar hue distributions.

On each trial, stimuli had a size of 3.67° × 2.75° and were randomly distributed on a non-visible 8 × 6 grid. The rows/columns of the grid were separated by a 2° empty space. The position of the stimuli within the grid was then slightly staggered (±1°) to create a less uniform appearance. The overall spatial layout and location of targets and distractors was generated independently on every trial.

### 2.3. Procedure

Stimuli were presented with PsychoPy 3 v. 2021.1.2 [34] on a 24-inch DELL P2419H monitor with a 60 Hz refresh rate and a resolution of 1980 × 1080 pixels. The experiment took place in a dimly lit room. The participants’ heads were kept stable with a chin and forehead rest positioned 57 cm away from the monitor.

**Figure 1.**
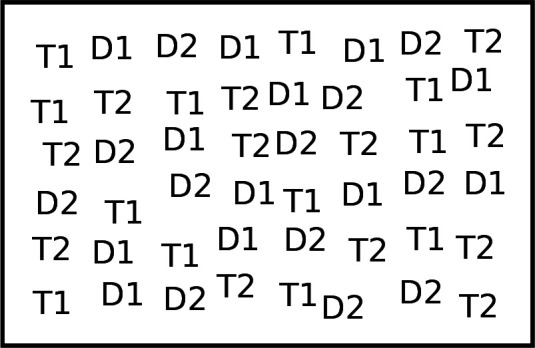
Visual foraging task. T1 and T2 represent the two target images, and D1 and D2 the two distractor images. In line with the recommendations of Lang *et al*. [7], we do not reproduce IAPS images in print. T1, T2, D1 and D2 correspond to the images used in the positive, neutral and negative blocks (interested readers may refer to the IAPS ID numbers listed in §2.2). The positions of targets and distractors were randomly and independently assigned on each trial.

As presented in figure 1, each trial involved 48 stimuli on a dark grey background. In three blocks of 16 trials each, participants had to select 12 instances of two target types (negative, positive or neutral images) and ignore 12 instances of two distractor types (neutral images). The three blocks differed by the set of images used for targets and distractors (i.e. they correspond to the positive, negative and neutral sets described in §2.2). All blocks were preceded by two training trials and were completed in one session of about 45 min. Block order was counterbalanced across participants.

On each trial, participants were instructed to select all the targets on the display as fast as possible, without selecting any distractor. They were free to select the targets in any order. Stimulus selection was triggered with mouse clicks, and when a target was selected, it disappeared. Distractor selection led to an error message and to the renewal of the trial until successful completion. When the trial was completed (i.e. all targets had been selected), a feedback screen appeared indicating the progression in the current block and the trial response time.

Each block began with the instruction screen, where target and distractor images were presented at the same size as in the experiment (3.67° × 2.75°). Then, participants were invited to press the space bar to display the four images in a bigger size (16.67° × 12.5°) for 8 s. This made sure that all participants identified the emotional images. They were then invited to start the training by pressing the space bar. After successful completion of the two training trials, the main experiment began with a new presentation of the images in 16.67° × 12.5° for 8 s, followed by the 16 test trials.

After successful completion of the three blocks of trials, participants were asked to complete the French version of the State-Trait Anxiety Inventory form Y (STAI-Y) [35] to measure trait anxiety. It consists of 20 statements evaluating anxiety experienced in general across various situations. Participants responded to each statement on a four-point Likert scale.

### 2.4. Data analysis

In accordance with previous studies (e.g. [24,31,33]), we assessed the foraging strategy with the number of runs for each trial and participant. A ‘run’ refers to the sequential selection of targets of the same category (i.e. same image). As such, with 24 targets divided into two categories, the number of runs varied between 2 and 12. The lower the number of runs, the fewer the switches between target categories, and vice versa.

Another indicator of foraging strategy was inter-target distance, which corresponds to the average distance in degrees of visual angle between two successive target selections in the same trial. Together, the number of runs and inter-target distance inform whether participants prioritize priming (i.e. they forage in runs and prioritize targets that share features with previous selections, even if they are further away) or proximity (i.e. they prioritize targets proximal to previous selections, even if it leads to more switches between target categories).

Another approach to measure foraging strategy consists of analysing foraging behaviour in a Bayesian multilevel model [36]. In Clarke *et al*.’s model, run behaviour is broken down into different biases that influence target selection, including a stick bias that measures the tendency to select targets in runs and a proximity bias that measures the tendency to select targets near to the last target selection. We applied this model to the current data and measured the stick and proximity biases to provide additional evidence for differing weights of priming and proximity in the selection balance [31] across the three emotional valence conditions.

Foraging performance was assessed with inter-target times, which correspond to the time that elapsed between two successive target selections in a given trial. We also assessed switch cost, which corresponds to the subtraction of inter-target times within runs from inter-target times between runs (i.e. it represents the cost, in milliseconds, of selecting targets from a different category as the previous selection when compared to the selection of targets from the same category as the previous selection).

Finally, the trait anxiety score of each participant assessed with the French version of the STAI-Y [35] was converted into a standard score (min = 1, max = 99, M = 50, s.d. = 10) using the conversion grid for adult participants.

The effects of target emotional valence on foraging strategy (number of runs, inter-target distance) and performance (inter-target time, switch cost) were assessed in an ANOVA with target emotional valence (positive, neutral, negative) as a within-subject factor. Moreover, the effect of trait anxiety as a continuous variable was also assessed in linear regression analyses with standard scores at the STAI-Y as the dependent variable.

Before performing the main analyses, trials with an average number of runs (0.08%), inter-target times (0.6%), inter-target distances (0.81%) or switch costs (0.56%) diverging from 2.5 s.d. of the individual mean were discarded from further analyses, for a total of 74/3600 (2.06%) discarded trials.

## 3. Results

### 3.1. Foraging strategy

**Figure 2.**
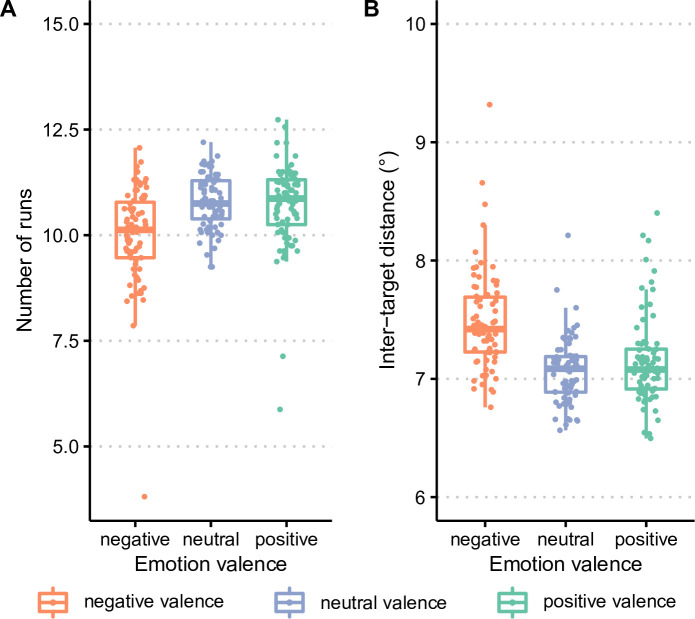
Foraging strategy as a function of target emotional valence. Results are shown for (A) the number of runs and (B) inter-target distance.

Results obtained on the number of runs are summarized in figure 2A. The ANOVA on the number of runs revealed a main effect of target emotional valence (F_2,148_ = 23.9, *p* < 0.0001, η_p_² = 0.24). The number of runs found in the negative-valence block (M = 10.0, s.d. = 1.2) was smaller than in the two other blocks (neutral-valence block: M = 10.8, s.d. = 0.6; positive-valence block: M = 10.7, s.d. = 1.0; both *p* < 0.0001 at Tukey honestly significant difference (HSD) post hoc tests). The number of runs, however, did not differ between the positive-valence and neutral-valence blocks (*p* = 0.81 at Tukey HSD post hoc tests). In other words, these results show that participants were more likely to select an image from the same category as the previous selection in the negative-valence block than in the neutral-valence and positive-valence blocks.

Results on inter-target distance, presented in figure 2B, also showed an effect of target emotional valence (F_2,148_ = 23.9, *p* < 0.0001, η_p_² = 0.24)*.* Average inter-target distance was higher for negative-valence targets (M = 7.6°, s.d. = 0.9°) than for neutral-valence (M = 7.1°, s.d. = .3°) and positive-valence (M = 7.2°, s.d. = .4°*)* targets (both *p* < 0.0001 at Tukey HSD post hoc tests). However, the difference between the neutral-valence and positive-valence blocks was not significant (*p =* 0.53 at Tukey HSD post hoc tests).

As described above, we also assessed foraging strategy with the Bayesian multilevel model described by Clarke *et al*. [36]. The analysis of the stick bias (values >0.5 indicate preference for making runs and selecting targets by category) showed a preference for foraging in runs in the negative block (stick bias = 0.67, 95% HDCI = 0.59–0.76), but not in the positive (stick bias = 0.45, 95% HDCI = 0.38–0.51) and neutral (stick bias = 0.45, 95% HDCI = 0.39–0.51) blocks. Moreover, the analysis of the proximity bias (higher values indicate a higher tendency to selecting the nearest target) indicated a weaker proximity bias in the negative block (proximity bias = 19.1, 95% HDCI = 18.4–19.9) compared to the positive (proximity bias = 22.1, 95% HDCI = 21.4–23) and neutral (proximity bias = 23.2, 95% HDCI = 22.5–24) blocks.

Altogether, both the ANOVA on classic foraging measures such as the number of runs and inter-target distance and the analysis with a Bayesian multilevel model [36], show that, during the negative-valence block, participants chose to forage in fewer runs and to select targets further away from the previous selection, compared to the neutral-valence and positive-valence blocks.

### 3.2. Foraging performance

**Figure 3.**
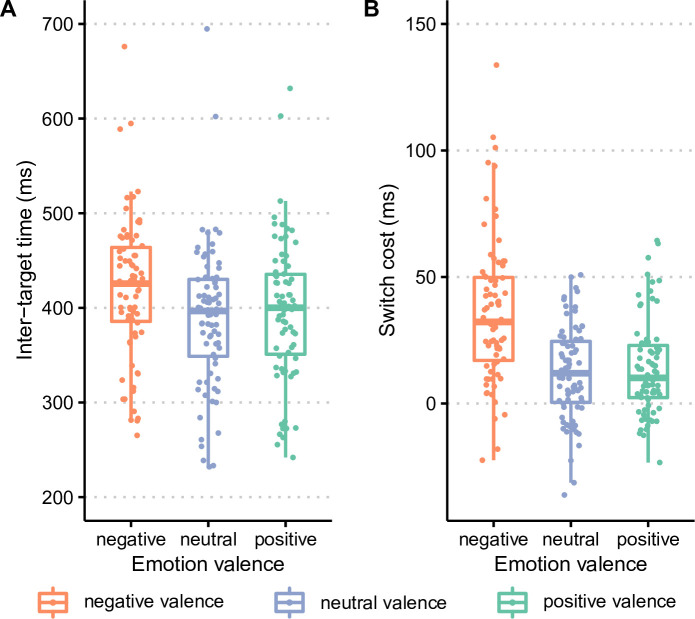
Foraging performance as a function of target emotional valence. Results are shown for (A) inter-target times and (B) switch costs.

The analysis of inter-target times, presented in figure 3A, revealed a significant effect of target emotional valence (F_2,148_ = 27.4, *p* < 0.0001, η_p_² = 0.27*)*, with higher inter-target times in the negative-valence block (M = 420 ms, s.d. = 75 ms) than in the neutral-valence (M = 389 ms, s.d. = 76 ms) and positive-valence (M = 396 ms, s.d. = 74 ms) blocks (both *p* < 0.0001 at Tukey HSD post hoc tests). Inter-target times did not differ between neutral-valence and positive-valence blocks (*p* = 0.22 at Tukey HSD post hoc tests).

As presented in figure 3B, the effect of target emotional valence was also found on switch costs (F_2,148_ = 28.7, *p* < 0.0001, η_p_² = 0.28), with higher switch costs for negative-valence targets (M = 36 ms, s.d. = 29 ms) than for neutral-valence (M = 12 ms, s.d. = 19 ms) and positive-valence (M = 14 ms, s.d. = 19 ms) targets (both *p* < 0.0001 at Tukey HSD post hoc tests). Switch costs, however, did not differ between positive-valence and neutral-valence targets (*p* = 0.85 at Tukey HSD post hoc tests).

Altogether, these results show that foraging performance was impaired in the negative-valence block compared with the positive-valence and neutral-valence blocks, leading to higher inter-target times and higher switch costs.

### 3.3. Trait anxiety

**Figure 4.**
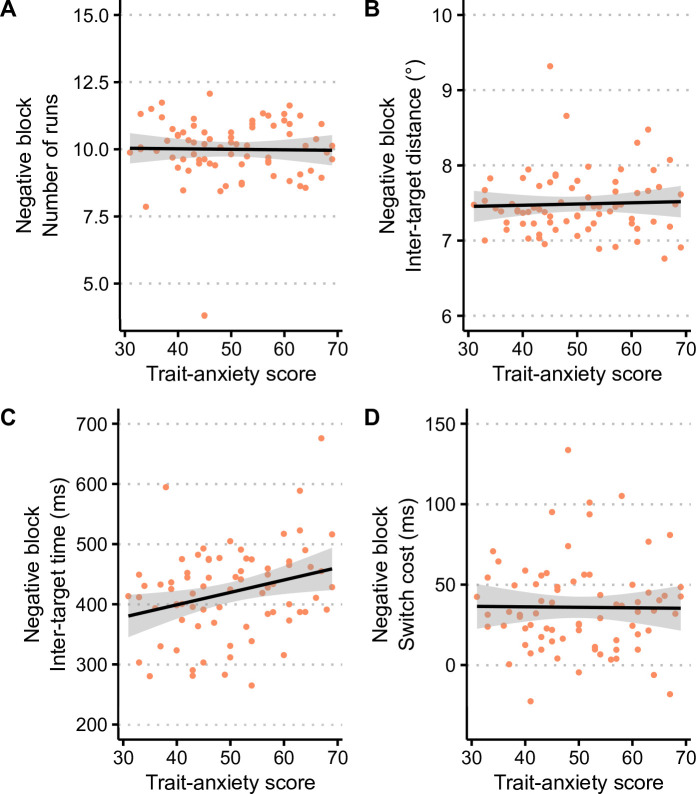
Relationship between trait anxiety score and (A,B) foraging strategy or (C,D) performance in the negative block. The black lines show linear fits with 95% confidence intervals.

To investigate the effect of participants’ trait anxiety, we examined the relationship between the standard scores at the STAI-Y (continuous measure of trait anxiety) and the measures of foraging strategy (number of runs, inter-target distance) and performance (inter-target time, switch cost). In this analysis, we focused on foraging behaviour in the negative-valence block, as it was expected to be related to trait anxiety level [11,22]. To do so, we conducted linear regression analyses with the foraging measures obtained in the negative block as dependent variables, and the STAI-Y standard score as predictor, separately for the number of runs, inter-target distances, inter-target times and switch costs. These analyses are presented in figure 4.

The analyses performed on measures of foraging strategy (number of runs in figure 4A, inter-target distance in figure 4B) did not reveal a significant relationship with STAI-Y standard scores (both *p* > 0.05 and both *R*² < 0.02*)*. Regarding foraging performance, STAI-Y standard scores did not significantly predict switch costs (*F* < 1, *R*² < 0.001, figure 4D), but the results showed a significant relationship between trait anxiety score and inter-target times (F_1,73_ = 6.3, *p* < 0.02, *R*² = 0.08*,* figure 4C) with the following model:

Inter-target times for negative targets = 2.06 * STAI-Y standard score + 316.8

Altogether, these regression analyses show that foraging strategy was not influenced by participants’ trait anxiety, but that foraging performance in the negative-valence block did depend on trait anxiety level, with increasing inter-target times as trait anxiety increases.

## 4. Discussion

In summary, the main finding of this study is that foraging for negative-valence targets leads to different foraging strategies and performance compared to foraging for neutral-valence or positive-valence targets. Indeed, the results on foraging performance, and notably on switch costs, show that switching between negative-valence targets was costlier than switching between neutral-valence and positive-valence targets. Importantly, the results on foraging strategy reveal that participants did adapt their foraging accordingly: they chose to forage in fewer runs during the negative-valence block compared with the neutral-valence and positive-valence blocks. Furthermore, we found that trait anxiety did not influence foraging strategy but influenced foraging performance for negative targets, with increased inter-target times for observers with high trait anxiety.

In accordance with the attentional bias towards emotional stimuli [8–10], we expected a difference in foraging strategy and performance between the emotional blocks and the neutral block. More precisely, we hypothesized poorer performance in the emotional blocks compared to the neutral block given that the multiple emotional targets in a trial may compete for current selection, while observers need to sequentially select them to complete the trial. Surprisingly, while we found the expected result in the negative-valence block compared with the neutral one, foraging behaviour in the positive-valence block did not differ from the neutral-valence block. Importantly, we did find a significant difference between the positive-valence block and the negative-valence block on all measures of foraging strategy and performance, even though images used in these two conditions were matched on arousal ratings. This suggests that the polarity of the valence (positive vs. negative) influenced foraging behaviour when arousal was controlled. However, it cannot be excluded that the effect of the negative stimulus compared to the neutral ones is also due to the difference in arousal between the two conditions, since negative stimuli were higher in arousal than neutral ones, which reflects the quadratic relationship between valence and arousal ratings [7]. Further studies are necessary to better understand the origin of this emotionality effect. Importantly, differing effects of positive and negative emotional images on single-target visual search performance have been reported by Bendall *et al*. [37] using a different procedure. In a single-target visual search task superimposed on an emotional image, the authors showed that reaction times were impaired for negative emotional images compared to neutral images, with no difference between the positive and neutral conditions, while accuracy revealed a different pattern of data. Therefore, the current results on inter-target times corroborate the findings of Bendall *et al*. [37], confirming that negative and positive images yield differing effects on reaction times, both in single-target visual search [37] and in visual foraging (current study). Future studies may investigate more closely the effects of emotional targets on visual foraging accuracy.

In a previous paper [31], we suggested that target selection during visual foraging was driven by the competition between target proximity (i.e. selecting targets proximal from previous selections), priming of features (i.e. selecting targets that share features with previous selections) and target value (i.e. selecting valuable or rewarding targets), which we named the ‘selection balance’. We notably showed that when one target type was associated with high value (e.g. number of points earned at target selection), the ‘selection balance’ was biased towards value and priming of features and against proximity. Here, we suggest that target emotional valence also contributes to the selection balance. Notably, it seems that negative emotional valence biases the selection balance towards priming of features, while neutral and positive emotional valences bias the selection balance towards spatial proximity from previous selections.

Previous research has shown that the number of runs is lower when task difficulty is high (e.g. feature vs. conjunction foraging [24,33]), but is larger when attentional load is increased (e.g. foraging and object tracking double-task [38]). Moreover, inter-target times are known to increase with task difficulty and attentional load [33,38]. Taken together with the current results, this suggests that foraging for negative-valence images is associated with fewer runs and higher inter-target times, and therefore with higher attentional load and task difficulty, than foraging for neutral and positive images.

Importantly, our results showed that as participants' trait anxiety scores increased, inter-target times on negative targets also increased. These findings are consistent with the attentional control theory [18], which suggests that individuals with high levels of trait anxiety exhibit impaired foraging performance when confronted with threatening stimuli. This impairment is assumed to occur because attentional resources that would typically be devoted to the task are instead consumed by anxious worry (see also [39,40]). This first evidence of a relationship between foraging performance and trait anxiety importantly suggests that visual foraging tasks involving emotional stimuli may be a useful tool for clinical psychologists as a potential therapeutic device in attentional bias modification (for similar discussions see [41,42]). It should, however, be noted that the relationship we found between trait anxiety and inter-target times is of small magnitude (*R*² = 0.08), and it may need to be replicated in later studies before conducting applied research in clinical populations.

Altogether, the current study importantly highlights that target emotional valence contributes to target selection during visual foraging and that it influences both foraging strategy and performance. Moreover, individual differences in foraging performance are related to individual characteristics such as trait anxiety, especially for negative targets. Previous studies in visual foraging have often involved simple stimuli such as coloured shapes (e.g. [24,31,43,44]), impeding any effect of target emotional contents. The current findings therefore have important implications for understanding visual foraging behaviour in ecologically valid conditions.

## Data Availability

All data and code are available on OSF [45].
